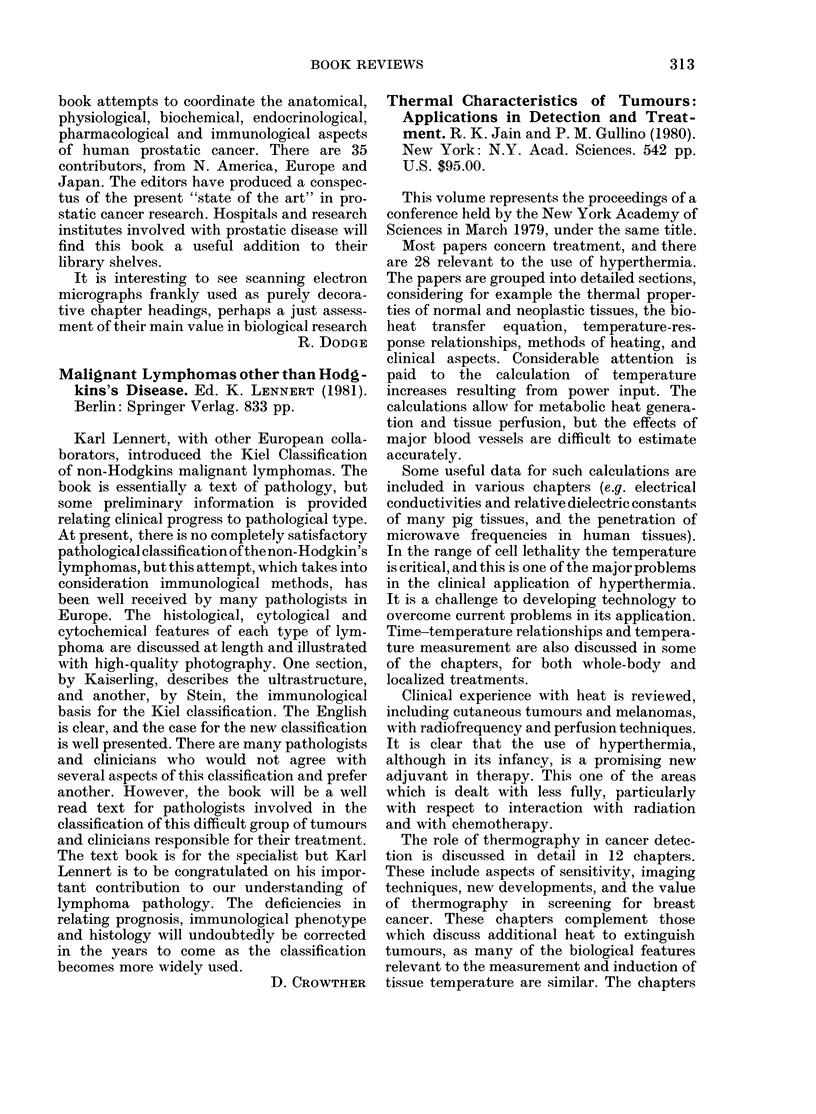# Malignant Lymphomas other than Hodgkins's Disease

**Published:** 1981-08

**Authors:** D. Crowther


**Malignant Lymphomas other than Hodgkins's Disease.** Ed. K. LENNERT (1981). Berlin: Springer Verlag. 833 pp.

Karl Lennert, with other European collaborators, introduced the Kiel Classification of non-Hodgkins malignant lymphomas. The book is essentially a text of pathology, but some preliminary information is provided relating clinical progress to pathological type. At present, there is no completely satisfactory pathological classification of the non-Hodgkin's lymphomas, but this attempt, which takes into consideration immunological methods, has been well received by many pathologists in Europe. The histological, cytological and cytochemical features of each type of lymphoma are discussed at length and illustrated with high-quality photography. One section, by Kaiserling, describes the ultrastructure, and another, by Stein, the immunological basis for the Kiel classification. The English is clear, and the case for the new classification is well presented. There are many pathologists and clinicians who would not agree with several aspects of this classification and prefer another. However, the book will be a well read text for pathologists involved in the classification of this difficult group of tumours and clinicians responsible for their treatment. The text book is for the specialist but Karl Lennert is to be congratulated on his important contribution to our understanding of lymphoma pathology. The deficiencies in relating prognosis, immunological phenotype and histology will undoubtedly be corrected in the years to come as the classification becomes more widely used.

D. CROWTHER